# Evaluation of maxillary sinus floor augmentation with the crestal approach and beta-tricalcium phosphate: a cone-beam computed tomography 3- to 9-year follow-up

**DOI:** 10.1186/s40729-020-00225-7

**Published:** 2020-07-10

**Authors:** Yoko Oba, Noriko Tachikawa, Motohiro Munakata, Tsuneji Okada, Shohei Kasugai

**Affiliations:** 1 Clinic for Implant Dentistry, Dental Hospital, Tokyo Medical and Dental University, 1-5-45 Yushima, Bunkyo-ku, Tokyo, Japan; 2 Implant Dentistry, Showa University Dental Hospital, Tokyo, Japan

**Keywords:** Graft material, Bone-added osteotome sinus floor elevation, Graft height, Radiographic analysis

## Abstract

**Background:**

We performed maxillary sinus floor augmentation using the crestal approach and beta-tricalcium phosphate (β-TCP) and a long-term evaluation using cone-beam computed tomography (CBCT).

**Methods:**

Twenty-three patients (30 implants) underwent sinus floor augmentation using the osteotome technique. Subjects underwent CBCT imaging before surgery, immediately after surgery, and at follow-up (≥ 3 years after surgery). We measured the changes in height of the augmented sinus floor (SL), the augmented bone above apex of the implant (BH), and the implant length that projected into the sinus (IL).

**Results:**

The mean SL decreased from 6.54 ± 1.51 (immediately after surgery) to 3.11 ± 1.35 mm (follow-up). The mean BH decreased from 3.17 ± 0.97 to − 0.25 ± 1.19 mm; the maxillary sinus floor in many implants was near the apex at follow-up. The SL at follow-up showed a strong correlation with the IL (*p* = 0.0057).

**Conclusions:**

Osteotome sinus floor elevation with beta-tricalcium phosphate was clinically effective. Cone-beam computed tomography analysis revealed that ≥ 3 years after surgery, the maxillary sinus floor was near the apex of the implant.

## Background

Alveolar bone is often vertically and horizontally absorbed after tooth extraction. In the maxillary molar region, pneumatization of the maxillary sinus progresses with tooth extraction [1, 2]. As the maxillary sinus floor approaches the alveolar crest, bone augmentation with maxillary sinus floor augmentation may be necessary when placing an implant in an atrophied maxillary molar region. Maxillary sinus floor augmentation is a surgical technique with high predictability, and it has been approximately 40 years since the lateral approach was first published by Boyne and James [3]. Summers [4] described the crestal approach, in which a tapered osteotome with a concave tip is used to enlarge the implant bore from the alveolar crest and elevate the sinus mucosa by fracturing the sinus base. Summers [5] also discussed bone-added osteotome sinus floor elevation (BAOSFE), in which the maxillary sinus floor is elevated by filling the implant bore with bone mass. The use of bone mass reduces the risk of sinus floor perforation and simplifies the elevation of the maxillary sinus floor and membrane [5].

In this study, we used beta-tricalcium phosphate (β-TCP) as the bone grafting material. Autogenous bone is also considered a bone grafting material as it has osteogenic, osteoinductive, and osteoconductive properties; however, possible complications of its use include invasion of the donor site and unregulated absorption of the graft material [6]. In recent years, maxillary sinus floor augmentation with various prosthetic bone materials has been introduced to reduce invasion, but the long-term prognosis of maxillary sinus floor augmentation with β-TCP and absorption changes in graft material is unknown. Although long-term evaluation has been investigated in numerous studies with panoramic radiographs, there are few reports on the use of cone-beam computed tomography (CBCT) for long-term evaluation. Issues that arise from using panoramic radiographs for evaluation include distortion, magnification, and an inability to perform cross-sectional and three-dimensional analysis. Problems with traditional medical CT include high radiation exposure and poor spatial resolution compared to CBCT. These problems have been resolved due to the development of CBCT, which allows for more accurate linear and volume measurements [7, 8].

In the present study, we performed maxillary sinus floor augmentation using the crestal approach and β-TCP as the bone grafting material. Clinical and long-term radiographic evaluations were performed using CBCT.

## Methods

### Subjects

**Fig. 1:**
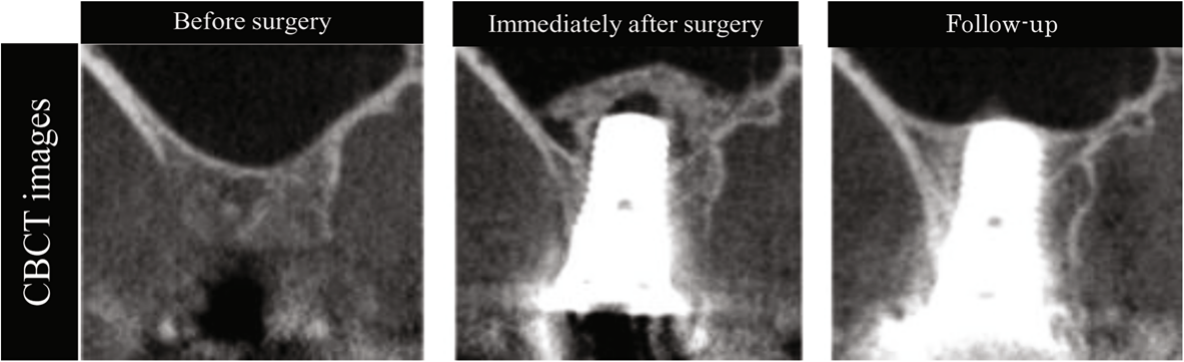
Treatment protocol for the present study. CBCT was performed before surgery, immediately after surgery, and at follow-up (≥ 3 years). CBCT, cone-beam computed tomography

This study included 23 patients (30 implants) who underwent maxillary sinus floor augmentation of maxillary defect sites using the crestal approach and β-TCP at the Clinic for Implant Dentistry, Dental Hospital, Tokyo Medical and Dental University from September 2007 to August 2014. All patients underwent CBCT imaging and examination before surgery, immediately after surgery, and at follow-up. To analyze the long-term changes in β-TCP, we performed a follow-up examination at 3 or more years after surgery when post-implant volume had stabilized [7] (Fig. 1).

### Patient selection

Patients were eligible for the study if they met the following criteria: (1) they had defects in the maxillary molar region and wished to recover occlusal function of the defect site with fixed prosthesis for implant; (2) they had well-established oral hygiene, without major dental issues or advanced periodontal diseases that could affect healing of the defects; (3) in preoperative coronal CBCT imaging, the distance from the alveolar crest of the site of implant placement to the maxillary sinus floor was ≥ 4 mm; (4) Schneiderian membrane thickness was < 5 mm.

Patients were excluded from the study if they met the following criteria: (1) were edentulous, due to the expected impact of the postoperative use of dentures; (2) required large-scale bone transplants or enlargement of bone width, such as onlay graft and guided bone regeneration, at the same time as maxillary sinus floor augmentation (crestal approach); (3) had an immune-related disease such as diabetes, osteoporosis, or rheumatoid arthritis; (4) were taking bisphosphonate; (5) had an underlying disease such as chronic maxillary sinusitis; (6) had otolaryngological problems; (7) had other unmanaged systemic diseases; or (8) were smokers.

Informed consent was obtained from all the patients regarding bone grafting material use, the surgical procedures, as well as the multiple CBCT scans. This study was performed with the approval of the Ethics Committee of Tokyo Medical and Dental University, Tokyo, Japan (approval number 309). Under patient-informed consent, the study was conducted in full accordance with the ethical principles of the Declaration of Helsinki. The study is compliant with the STROBE guidelines.

### Surgical methods

**Fig. 2:**
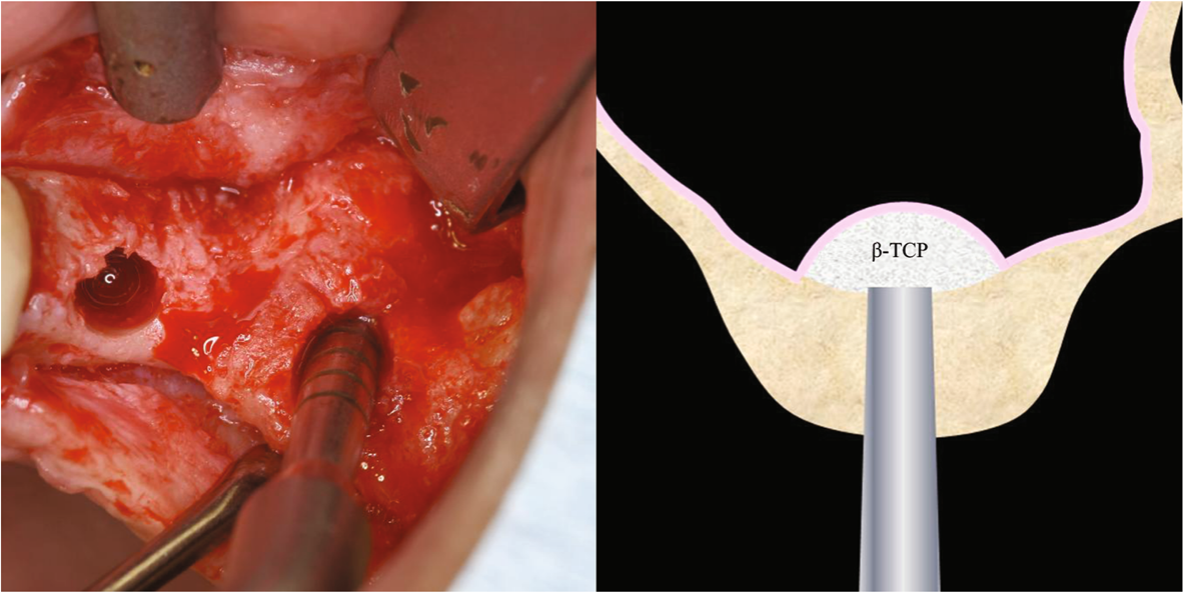
Maxillary sinus floor augmentation using the crestal approach followed by bone-added sinus floor elevation (BAOSFE)

After preoperative examination, subjects underwent maxillary sinus floor augmentation using the crestal approach at the same time as implant placement (BAOSFE). Maxillary sinus floor augmentation was performed by expert surgeons. Patients received local anesthesia (2% lidocaine hydrochloride with 1/80,000 epinephrine: 2% Xylocaine Dentsply Sirona) and systemic management. We performed an alveolar crest incision at the buccolingual center of the defect, and the mucoperiosteal flap was detached. Using the designated osteotome for implant bore formation, we hammered the cortical bone of the maxillary sinus floor from the direction of the alveolar crest until fracture and used the osteotome to slowly fill the cavity with β-TCP presoaked in physiological saline solution to elevate the maxillary sinus membrane, after which we placed the implant. To prevent infections, postoperative antibiotics *and* anti-inflammatory analgesics were prescribed 3 times per day for 1 week. Disinfection with mouthwash was indicated 3 times per day for 2 weeks. Stitches were removed after approximately 2 weeks. Patients were checked once per month to evaluate postoperative healing, and prosthetic treatment was initiated 6 months after surgery (Fig. 2).

### Selecting the implant body

We selected the length of the implant body by determining the thickness of the existing bone, planning for an elevation of 3.5–4.0 mm. For single dental defects, we used implants that were 9.5–10 mm in length. When there were continuous defects in multiple teeth and placement of multiple implants and upper structures to connect these implants was planned, we also used implants 8 mm in length. We did not use short implants < 8 mm in length.

### Bone grafting material

We used OSferion G1 (diameter, 0.5–1.5 mm; Olympus Terumo Biomaterials Corp., Tokyo, Japan), which is β-TCP with a biphasic crystal structure. OSferion is a β-TCP product with high purity produced by a mechanochemical method. It has a porosity of 75% and contains macropores with pore diameters of 100–400 μm and micropores with diameters ≤ 1 μm. It is suited for vascular neogenesis and promotion of surface absorption.

### Radiographic examination

We used CBCT to perform examinations at each stage of every case, including before surgery, immediately after surgery, and at follow-up (Fine Cube; Yoshida, Tokyo, Japan. voxel size 0.156 × 0.157 × 0.146 mm). We used dental CBCT for X-ray CT because of its superior spatial resolution. The measurement of the linear variables was performed on CBCT coronal cross-sections parallel to the longitudinal direction of the implant. The following are explanations of the linear variables:

- Height of the augmented bone above the apex of the implant (BH): measured as the distance from the intercept of the long axis of the implant and the lower edge of the maxillary sinus membrane to the apex of the implant.
- Height of the augmented sinus floor (SL): calculated as the sum of the IL and BH (SL = IL + BH).
- Implant length (IL): the length of the part of the implant body that projected into the maxillary sinus, measured as the mean distance from the apex of the implant to the buccal and palatal sides of the floor of the maxillary sinus
- Residual bone height (RBH): the long diameter of the existing bone to the maxillary sinus at the implant site, measured as the mean distance from the crown to the buccal side or palatal side of the floor of the maxillary sinus.

**Fig. 3:**
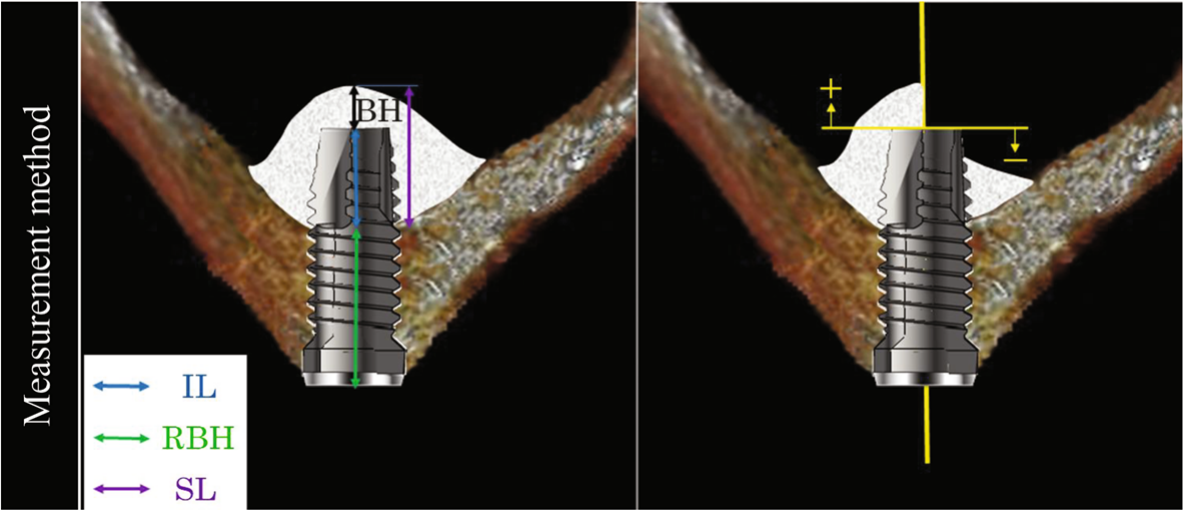
Measurement method using CBCT. Radiographic examination on the coronal cross-section images parallel to the longitudinal direction of the implant. The linear variables include height of the augmented bone above apex of the implant (BH), height of the augmented sinus floor (SL), implant length that projected into the sinus (IL), residual bone height (RBH)

When there was augmented bone above the apex of the implant, BH was defined as the distance from the intercept of the extended central axis of the implant body and the lower edge of the maxillary sinus membrane to the apex of the implant (+ mm). If absorption of the augmented bone advanced beyond the apex of the implant to the crown side, BH was defined as the distance from the apex of the implant to the line that connects the most apical contact between the implant and the augmented bone on the buccal and palatal sides along the central axis of the implant body (mm) (Fig. 3). The measurements were performed by 3 examiners (dentists), and the mean value was used as the clinical data.

### Statistical analysis

We compared the vertical dimensions of the augmented bone around the implant immediately after surgery (BH immediately after surgery) and at follow-up (BH at follow-up) and analyzed the changes using a paired *t* test. In addition, we statistically analyzed the impacts of the observation period, RBH, BH immediately after surgery, IL, and SL immediately after surgery on the decrease in the BH volume from immediately after surgery to follow-up (volume of BH decrease) using Spearman’s correlation test. Similarly, we determined if the observation period, RBH, BH immediately after surgery, IL, and SL immediately after surgery affected the volume of maxillary sinus floor augmentation at follow-up (SL at follow-up).

## Results

### Participants

This study included 23 patients (30 implants). The patients included 7 men and 16 women with a mean age of 58.1 ± 12.7 (24–75) years. The mean observation period from implant placement surgery to follow-up was 81.9 ± 25.1 (36–112) months (Table 1). The implant characteristics are shown in (Table 2). Of 30 placed implants, 22 were connected crowns, including 4 implants 8 mm in length, and 8 single crowns. Regarding locations, 12 implants were inserted at the premolar area, and 18 were at the molar area.

**Table 1:** Observation period

Observation period (months)	Number of implants
36~47	4
48~59	4
60~71	1
72~83	4
84~95	2
96~107	12
108~119	3
AVG	81.9
SD	25.1
Min	36
Max	112

**Table 2:** Implant characteristics

Implant type	Diameter (mm)	Length (mm)	Number
Straumann	3.3	10	1
	4.1	10	2
	4.8	8	1
		10	5
Nobel Biocare	4.0	10	9
Xive	3.8	8	3
		9.5	6
	4.5	9.5	2
	5.5	9.5	1
Total			30

All 30 implants achieved osseointegration. There were no complications during the observation period.

### Radiographic evaluation

**Fig. 4:**
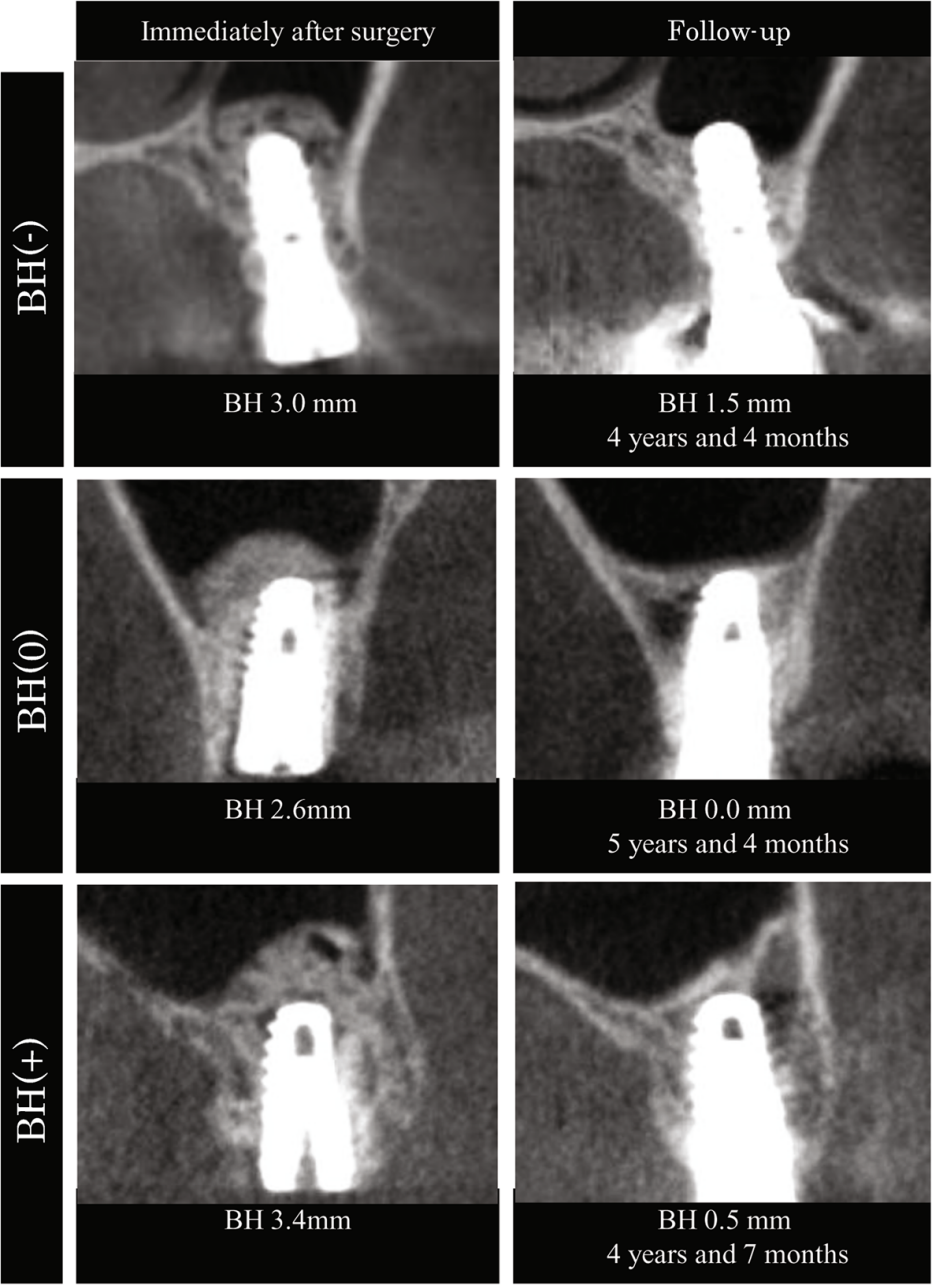
CBCT images immediately after surgery and at follow-up. Immediately after surgery, the maxillary sinus floor was above the implant apex of all implants. At follow-up, the maxillary sinus floor was below the implant apex (BH < 0), at the same height (BH = 0), or above implant apex (BH > 0). BH, augmented bone above apex of the implant

On CBCT images, preoperative membrane thickness was 1.09 ± 0.64 (0.28–2.58) mm. β-TCP and existing bone could be identified immediately after surgery, but the boundary between them was not visible at follow-up. Although β-TCP formed a dome above the implant apex immediately after surgery, at follow-up, the area of augmented bone had decreased (Fig. 4).

For each implant, we measured the BH immediately after surgery, BH at follow-up, SL immediately after surgery, SL at follow-up, IL, and RBH (Table 3).

**Table 3:** Measurement results from CBCT images

	BH immediately after surgery	BH at follow-up	SL immediately after surgery	SL at follow-up	IL	RBH
Mean (mm)	3.17	− 0.25	6.54	3.11	3.37	5.68
SD (mm)	0.97	1.19	1.51	1.35	1.18	1.13
Minimum (mm)	1.20	− 2.58	3.24	0.40	0.90	4.00
Maximum (mm)	5.50	3.00	8.70	5.57	5.50	8.06

### Decrease in BH

The mean BH immediately after surgery was 3.17 ± 0.97 (1.20–5.50) mm in the direction above the implant apex. However, in all cases, the BH decreased at follow-up. The mean BH was − 0.25 ± 1.19 (− 2.58–3.00) mm, which was significantly different to the BH immediately after surgery (***p = 1.629E−13). The mean decrease in BH between measurements taken immediately after surgery and those obtained at follow-up was 3.42 ± 1.43 (0.50–6.80) mm.

**Fig. 5:**
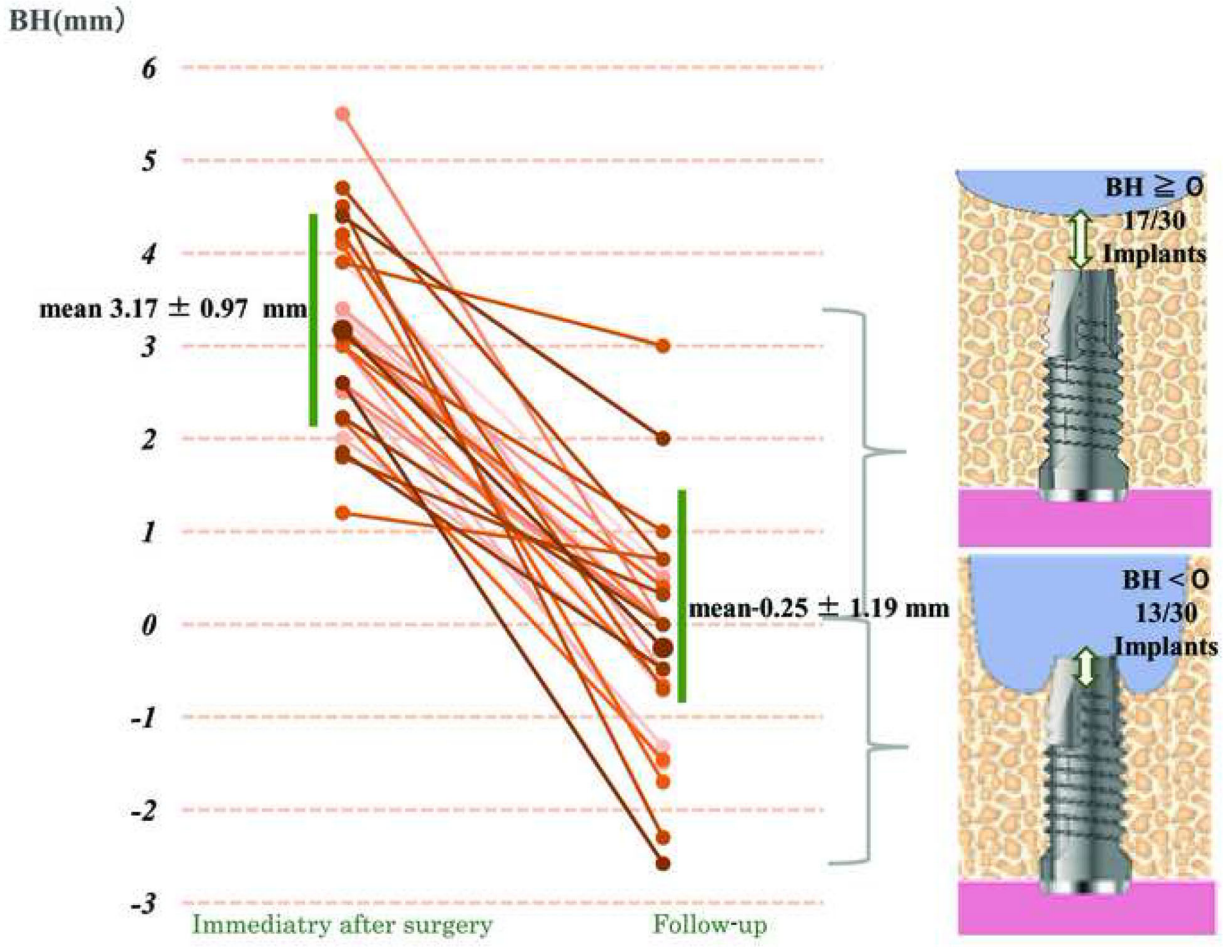
Changes in BH in 30 implants. The mean BH immediately after surgery was 3.16 ± 0.97 (1.2–5.5) mm, while the mean BH at follow-up was 0.25 ± 1.19 (− 2.58–3.00) mm; a mean decrease of 3.42 ± 1.43 (0.50–6.80) mm. After surgery, the maxillary sinus floor was below the implant apex (BH < 0) in 13 of 30 implants. BH, augmented bone above apex of the implant

At follow-up, BH approached zero in many implants, and the position of the maxillary sinus floor became consistent with the implant apex in many cases. BH at the follow-up period became negative, and bone could not be confirmed around the implant apex in CBCT images in 13 of 30 cases (Fig. 5). No clinical problems were observed in any of the cases regardless of whether the BH values were positive or negative at follow-up.

### IL, RBH, and SL

The mean IL immediately after surgery in CBCT images was 3.37 ± 1.18 (0.90–5.50) mm. The mean RBH was 5.68 ± 1.13 (4.00–8.06) mm. The mean SL immediately after surgery was 6.54 ± 1.51 (3.24–8.70) mm, and the mean SL at follow-up was 3.11 ± 1.35 (0.40–5.57) mm (Table 3).

The intraclass correlation coefficient (ICC [1, 2]) was calculated based on a binary variate model and then evaluated as an index of reproducibility between 3 evaluators. Since a high inter-evaluator reproducibility (ICC [1, 2] > 0.9) was judged for all the evaluation items, the reliability of measurement could be judged as high. Inter-evaluator reproducibility: calculation of the intra-class correlation function.

### Statistical analysis

#### Impact of each factor on BH decrease

The decrease in BH from immediately after surgery to the follow-up examination showed a strong correlation with the BH immediately after surgery (***p = 0.0034) and the SL immediately after surgery (***p = 0.00009), and was also correlated with the IL (*p = 0.0283). However, there was no correlation with the observation period or the RBH (Table 4).

**Table 4:** Impact of each factor on the observed decrease in BH

	*r*	*p* values	
Observation period	0.1060	0.5778	
RBH	0.1220	0.5192	
BH immediately after surgery	0.5173	0.0034	***
IL	0.3970	0.0283	*
SL immediately after surgery	0.6527	0.00009	***

Spearman’s correlation test (two-tailed); *p < 0.05; ***p < 0.005

#### Impact of each factor on SL at follow-up

The SL at follow-up showed a strong correlation with the IL (*p* = 0.0057) and was also correlated with the SL immediately after surgery (*p* = 0.0170). However, there was no correlation with the observation period, RBH, or BH immediately after surgery (Table 5).

**Table 5:** Impact of each factor on SL at follow-up

	*r*	*p* values	
Observation period	0.1310	0.4888	
RBH	0.2900	0.1201	
BH Immediately after surgery	0.1475	0.4367	
IL	0.4926	0.0057	**
SL Immediately after surgery	0.4320	0.0170	*

Spearman’s correlation test (two-tailed); *p < 0.05; **p < 0.01

## Discussion

The survival rate for the maxillary sinus floor augmentation crestal approach according to Del Fabbro et al. [9] was 98.12% at 1 year, 97.40% at 2 years, 96.75% at 3 years, and 95.81% at 5 years. Similar to the lateral approach, it has a high predictability and is widely used in clinical settings. The advantage of the crestal approach is that the surgery is noninvasive and more rapid than the lateral approach. However, a disadvantage of this method is that as the surgery is performed blind, technical expertise is essential. Moreover, the amount of elevation is limited and any response to perforation of the sinus membrane is difficult.

### Membrane thickness

In this study, patients with a history of maxillary sinusitis, those with suspected dental maxillary sinusitis such as apical lesions that cause mucosal thickness, those suspected of having allergies such as hay fever, those having findings considered to be mucocele, and those with a mucosal thickness ≥ 5 mm were excluded. Regarding the thickness of the maxillary sinus membrane, many reports state that the physiological thickness is 1–2 mm [10]. The preoperative maxillary sinus membrane thickness in this study was 1.09 mm on average, which is equivalent to the value (average 1.17 mm: CT 1.33 mm: histology 0.48 mm) in the report on the physiological thickness of the maxillary sinus membrane using cadaver and CT [10]. There is concern that increase in the thickness of the maxillary sinus membrane is associated with bacterial infection and a decrease in elimination function. However, there are also maxillary sinuses where even if the sinus membrane thickens, ventilation of the mucosal epithelium and the elimination function remain normal. In addition, there is no data or guideline that dentists and otolaryngologists alike can use regarding the relationship between mucous membrane thickness and decreased maxillary sinus function [11]. Even when there is mild mucosal membrane thickening, we suggest that there will be no problem with ventilation and the elimination function provided there is no clinical symptom and the maxillary sinus ostium is patent. In a preoperative analysis by CBCT, Shanbhag et al. [12] considered sinus membrane thickness ≥ 2 mm pathological, and when it was ≤ 2 mm, ostium patency was not classed as obstructed. Even when the thickness was 2–5 mm, impairment was seen in only 6.7% of cases while increased findings of ostium obstruction were reported in 24% and 35.3% of cases when the thickness was > 5 mm and > 10 mm, respectively. However, a mucosal thickness > 2 mm was seen in 60.6% of patients, and when a thickness of ≤ 2 mm was set as normal, there seemed to be an increase in patients being falsely diagnosed with maxillary sinusitis. Carmeli et al. [13] examined thickness of the mucous membrane and ostium obstruction by CT. They reported that membrane thickness exceeding 5 mm is associated with the risk of ostium obstruction. Therefore, in this study, in addition to the medical history and the presence or absence of clinical symptoms such as nasal congestion, rhinorrhea, and headache, patients whose CBCT examination revealed mucosal membrane thickness below 5 mm, and the ostium was not affected by edema, were the subjects.

### Length of the implant

Short implants were recently introduced owing to improvements in the implant surface. The European Association for Osseointegration (EAO) 2006 Consensus Conference defines short implants as those ≤ 8 mm in length. A systematic review based on meta-analyses through randomized comparison tests showed that the 5-year survival rate of implants ≤ 8 mm in length was not significantly different compared to longer implants [14]. However, when using implants ≤ 8 mm in length, the superstructure was connected. In addition, Renouard and Niasand [15] reported that although there is a higher tendency of failure in short implants, this trend decreases with a rough surface. Manzano et al. [16] reported that as the maxillary molar region is fragile bone, implants < 10 mm in length have a higher risk of decreased survival. Since there is insufficient data on the long-term survival of non-connected implants < 10 mm in length in the maxillary molar region, in this study, we used implants 9.5 to 10 mm in length for single crown restorations, and implants 8 mm in length were connected to the superstructure. Implants < 8 mm in length were not used.

### Residual bone height

The use of the crestal approach is limited to cases in which the vertical volume of the existing bone allows initial fixation of the implant. A prospective study by Zitzmann and Schärer [17] recommended that the existing vertical bone dimension at the implant site should be at least 6 mm. Pjetursson et al. [18] compared the prognosis of implants placed by the osteotome technique and those place by the conventional method and reported that the osteotome technique has a good prognosis if the vertical dimension of the existing bone is ≥ 5 mm. Călin et al. [19] noted in a systematic review of the osteotome technique that if the vertical dimension of the existing bone is ≥ 4 mm, the success of the implant is not affected, but below that value, there is an impact. In this study, we obtained a good initial fixation in all cases with a mean bone vertical dimension of 6.35 ± 1.15 (4.2–8.5) mm, and there were no clinical problems in any implant at follow-up.

### β-TCP absorption and new bone formation

The β-TCP used in this study has only osteoconductive properties during absorption. Okada et al. [20] reported that in maxillary sinus floor augmentation with the lateral approach, β-TCP was slowly replaced by new bone during the year after the graft was received. In this study, dome-shaped β-TCP was observed immediately after surgery, and the CBCT image decreased at follow-up. The boundary between β-TCP and existing bone that could be identified immediately after surgery was not visible during long-term observation, confirming absorption of the β-TCP and remodeling through maxillary sinus floor augmentation using the crestal approach.

### BH and SL

The BH was 3.17 ± 0.97 (1.20–5.50) mm immediately after surgery, and − 0.25 ± 1.19 (− 2.58–3.00) mm during the follow-up period of 81.9 ± 25.1 (36–112) months after surgery, with a decrease of 3.42 ± 1.43 (0.50–6.80) mm. A CBCT analysis of maxillary sinus floor augmentation using the lateral approach with β-TCP by Okada et al. [7] revealed a decrease in volume at the implant site 6 months to 1 year after surgery, which stabilized after 3 years. Zijderveld et al. [21] examined the prognosis of maxillary sinus floor augmentation using the lateral approach with autogenous bone and β-TCP with panoramic radiography and reported that in both cases, most absorption occurred during the first 7.5 months, with only small changes observed after 1.5 years. At the follow-up examination in this study, volumetric changes following grafting had already stabilized, and the BH in many implants at this point was approximately zero. This indicated that over time, the position of the maxillary sinus floor approached the implant apex.

BH loss at follow-up was negative in all of the implants, but we were able to obtain good long-term prognosis regardless of whether the BH values were positive or negative at follow-up. Okada et al. [7] also reported that there were no clinical problems in maxillary sinus floor elevation using the lateral approach with β-TCP alone, regardless of the position of the maxillary sinus floor relative to the implant apex. Nedir et al. [22] also reported that in graftless maxillary sinus floor augmentation using the crestal approach, long-term prognosis was good if the implant apex protruded into the maxillary sinus.

The crestal approach for maxillary sinus floor augmentation elevated the maxillary sinus floor less than the lateral approach. Zitzmann and Schärer [17] reported that with the lateral approach, the volume after surgery was 10 mm when the implant was placed simultaneously and 12.7 mm when the implant was placed later, whereas it was 3.5 mm with the crestal approach. Pjetursson et al. [23] reported that maxillary sinus floor augmentation with the crestal approach using deproteinized bovine bone mineral (DBBM) resulted in a mean elevation of 4.2 mm at an average of 3.2 years after surgery. In this study, the mean volume of maxillary sinus floor elevation immediately after surgery was 6.54 ± 1.51 (3.24–8.7) mm and 3.11 ± 1.37 (0.40–5.57) mm at follow-up.

The most common intraoperative complication of maxillary sinus floor augmentation with the crestal approach is perforation of the sinus membrane [15]. It is difficult to treat, but Pjetursson et al. [23] reported that it occurs in 10.4% of cases, whereas Călin et al. [19] reported a mean of 6.28% (0–26%). While stable elevation of the maxillary sinus is desired for good implant prognosis, intraoperative elevation of the maxillary sinus floor must be minimized to avoid perforation of the sinus membrane. In this study, there was a strong correlation between the SL at follow-up and the IL; there was also a correlation with SL immediately after surgery. However, there was no correlation with BH immediately after surgery. As such, to secure long-term maxillary sinus floor elevation, the length of the implant protruding into the maxillary sinus is important. Si et al. [24] performed maxillary sinus floor augmentation using the crestal approach and reported that the volume of elevation of the maxillary sinus floor is related to the length of the implant protruding into the sinus. In addition, there was a strong correlation between the decrease in the BH from immediately after surgery to follow-up and both the SL immediately after operation and the BH immediately after operation; there was also a correlation with the IL. Therefore, increased maxillary sinus floor elevation also increases absorption; the material above the implant apex tends to be absorbed.

It is not effective to elevate the maxillary sinus floor much beyond the implant apex considering absorption, and it should be kept within an appropriate range to avoid perforation of the sinus membrane. There are few studies of the appropriate elevation volume of the maxillary sinus floor in maxillary sinus floor augmentation with the crestal approach. Pjetursson et al. [18] compared maxillary sinus floor elevation using the crestal approach with DBBM and without a graft. With a graft, there was a dome after surgery similar to that in the present study. This site gradually shrank, reducing the maxillary sinus floor elevation to 4.1 mm 3 years after surgery. However, cases without a graft showed slight structural changes between the implant apex and maxillary sinus floor membrane immediately after surgery, and the maxillary sinus floor elevation 3 years after surgery was 1.7 mm. As such, maxillary sinus floor elevation can be secured with higher predictability by using a graft.

Si et al. [25] compared the use of a graft of a mixture of DBBM and autogenous bone to cases that did not receive a graft and reported that elevation was significantly higher 6 months after surgery in cases that received a graft, but decreased thereafter. Three years after surgery, the elevation in each case was approximately 3 mm. Thus, future studies should be conducted to clarify the optimal elevation volume of the maxillary sinus floor and selection of graft material in maxillary sinus floor augmentation with the crestal approach. Sonoda et al. [26] surveyed the relationship between the volume of transplanted bone and the volume of maxillary sinus floor elevation using the crestal approach. In blind maxillary sinus floor augmentation using the crestal approach, if the membrane perforation risk is reduced by measuring the transplant material suited for the volume of maxillary sinus floor elevation needed, the success rate of the crestal approach may be increased.

This study has some limitations. First, the size of the study population is small. Further, other clinical parameters, such as sinus width and the type of defect (single or continuous) on which the crestal approach was applied, might affect the height of the augmented bone. In this study, these parameters were not taken into account. Therefore, these issues should be addressed in future studies.

## Conclusion

To conclude, we have shown that maxillary sinus floor augmentation using the alveolar crest approach with beta-tricalcium phosphate is a clinically effective treatment. In addition, cone-beam computed tomography analysis showed that beta-tricalcium phosphate remodeled the site ≥ 3 years after surgery, as the boundary with the existing bone was not visible. Moreover, the position of the maxillary sinus floor in many implants stabilized near the apex of the implant.

## Data Availability

The datasets used and/or analyzed during the current study are available from the corresponding author on reasonable request.

## Abbreviations

β-TCP: Beta-tricalcium phosphate
CBCT: Cone-beam computed tomography
